# Increasing the inclusivity of digital health co-production: an integrative review

**DOI:** 10.3389/fdgth.2025.1636469

**Published:** 2025-10-17

**Authors:** Ian Litchfield, Gayathri Delanerolle, Helen Juffs, Stephanie Bloxham, Sian Dunning, Lorraine Harper

**Affiliations:** ^1^ Department of Applied Health Sciences, University of Birmingham, Birmingham, United Kingdom; ^2^ Birmingham Health Partners, Birmingham, United Kingdom; ^3^ Birmingham Voluntary Services Council, Birmingham, United Kingdom; ^4^ West Midlands Health Technology Innovation Accelerator, Birmingham, United Kingdom; ^5^ University Hospitals Birmingham NHS Foundation Trust, Birmingham, United Kingdom; ^6^ Medical Devices Testing and Evaluation Centre, UHB NFT, Birmingham, United Kingdom

**Keywords:** digital health, digital inclusion, co-production, health inequalities, patient engagement

## Abstract

Co-production is increasingly being used to develop sustainable improvements in health service delivery that are shaped by the experiences and needs of a diverse range of stakeholders including patients and healthcare providers. The process also offers a compelling means of fundamentally addressing the key issues of acceptability and applicability of digital health tools that contribute to ongoing inequity in the use of digital health technologies. However, creating and moderating hybrid digital health co-production teams is hindered by heightened obstacles to inclusivity and equitability of the cost and complexity of digital healthcare, and the diverse digital experience amongst the relevant stakeholders. With previous examples of co-production that involve direct interaction between developers and diverse groups of patients and staff rare, this integrative review has collated the latest evidence on engaging these diverse stakeholders in healthcare innovation, with best practice in co-production, and presents it within a framework representing the five core steps of co-production: *Set-up, Discovery, Definition, Development,* and *Delivery*. This guidance includes structured and tailored training in co-production and the concepts of digital health, surfacing and challenging existing assumptions around data security and confidentiality, defining funding models, introducing and refining protypes of increasing sophistication, and structured implementation and evaluation of both the co-production process and its outputs.

## 1 Introduction

The capability of digital health technologies to automate and streamline effective and equitable care is recognised globally and they are beginning to transform the way medical professionals deliver care and patients manage their health in a range of settings and locations (1). However, the shift towards digitally enabled health care is a complex process involving technologies of varying functionality and purpose, and incorporating significant changes to pathways, workflows, patient engagement, and broader systems of delivery (2, 3). Implicit within this digital transformation is that relevant technologies are available and applicable to all levels of society, yet discrepancies exist in the extent to which patients access and utilise digital health technologies (4), where it is impacted by their affordability (5) and patients varying levels of confidence and sophistication (6). These differences are compounded by the growing sophistication in the functionality of devices and the infrastructure they require meaning that underserved populations (which we define here as those who are economically deprived and/or from ethnic minorities that are engaged less effectively by formal healthcare interventions (7), frequently miss out on the comparative advantages of digital health afforded those that are better educated or of higher socio-economic status (8). This divide in the access and utilisation of digital health technologies have multiple and widely understood social determinants relating to resource, education, ethnicity, digital literacy, and connectivity (9). There are a number of ways these issues might be addressed including the provision of free data, hardware, and tailored training (10), amongst which and arguably the most fundamental, is to ensure that the design of any digital health tool or solution is directly compatible with the diverse range of patients its intended to serve (11). In other areas of healthcare, the needs of a diverse range of stakeholders including patients and healthcare providers have been successfully accommodated through the use of co-production (12, 13). The process of co-production has multiple definitions, with some 60 being noted in a recent review which recommended that future work should instead of being caught up in agreeing on the precise definition instead focus on the shared core principles of co-production (14). Therefore for the purposes of this review we define it as the process or methodology that encourages participants to identify a problem before empowering them to solve it, an iterative process involving open and equitable interaction between service users and those involved in producing or providing a service (15).

There is growing evidence of the benefits of co-production in developing digital health solutions (16–18). The success of co-production is predicated on transparent communication (19), the mutual exchange of knowledge (20), and equitability of decision-making authority (21). However, co-production is vulnerable to a number of challenges associated with the ability and opportunity to participate and the accommodation of stakeholders from necessarily diverse backgrounds (22). This means that although the potential of co-production is widely understood, its practical application often falls short, leading to power imbalances amongst stakeholders, the tokenistic involvement of patients, and co-produced solutions that lack sustainability (23). This is particularly true of co-production in digital health where obstacles to inclusivity and equitability are heightened by the cost and complexity of digital healthcare, and greater diversity of experience in digital technologies amongst the relevant stakeholders including diverse and underserved populations, health care staff of various role and responsibility, and technology developers (12, 19, 24, 25).

There is growing recognition that more robust strategies are needed to pursue inclusive digital co-production, though there is little specific evidence to draw on (25–29). There are though lessons that might be learnt from combining successful strategies for engaging diverse patient populations in health and care improvement initiatives with the latest evidence of effective co-production (22, 30). This review collates these two strands of evidence, presenting them within the five core steps of co-production. In this way we provide practicable insight into how the challenges to inclusive digital co-production can be addressed.

## 2 Methods

The work consists of an integrative review of research relating to inclusive co-production activities in (digital) health that includes best practice and latest evidence of optimum engagement activities with a range of stakeholders including underserved populations (31). The intention was not to identify every piece of work that has been conducted in co-design and -production, but to follow best practice in conducting integrative evidence reviews, summarizing the empirical and theoretical literature illustrated by recent and relevant examples to map this evidence against the five core steps of the co-production process as outlined in Section 2.2 (32).

Ultimately, we describe the challenges to inclusive co-production, and where possible the measures that might be taken to mitigate them. Study eligibility criteria were established using the Population, Intervention, Comparison, Outcome, and Study design (PICO) framework (33) (see Table 1) and we have described our search in accordance with the Preferred Reporting Items for Systematic Reviews and Meta-Analyses (PRISMA) checklist (34).

**Table 1:** Summary of study eligibility.

Type of study	Population or Problem	Intervention or Exposure	Comparison	Outcome
Primary research drawing on a range of methodologies including, qualitative studies, and mixed methods. Reviews of various design, and a variety of grey literature, including commentaries, conference papers, and regulatory guidance	Adherence, and engagement with co-production in digital health innovation amongst individuals from underserved communities, technology developers and healthcare staff	Elements or initiatives developed or adapted to improve access, adherence and/or engagement and completion in any of the five core steps of co-production	Usual or routine co-production activities	A range of inclusive or mitigative co-production related techniques to optimise engagement and output of co-production

### 2.1 Search strategy

The literature was searched in June 2025 from 2000 onwards for recent examples and evidence of best practice in co-production or otherwise engaging a diverse range of stakeholders in health-related innovation. This timespan allows us to describe recent research relevant to current models of (digital) co-production and the latest understanding of the challenges to inclusive co-production. We created a search for one database and adapted it for use in the others used the following electronic medical databases: MEDLINE, and PubMed, and supplemented by citation searches and hand searches of including of Google Scholar. The inclusion criteria for our review comprised primary research that were peer-reviewed and relevant grey literature including regulatory guidance, only work published in English was considered. The search terms can be found in Supplementary File 1.

### 2.2 Data extraction and synthesis

The data was extracted and placed against the five core steps of co-production by the first author in discussion with the third author. A primarily narrative approach consistent with the recommended analytical method for narrative synthesis was used to summarise the nature and effect of the evidence for inclusive co-production within the five steps (31). The criteria for selecting the included work were based on their relevance to the design and delivery of future inclusive digital co-production activities. We extracted data that included (i) programme overview (ii) author and publication date (iii) nature of evidence (iv) country of origin (v) summary of recommendations.

## 3 Results

**Figure 1:**
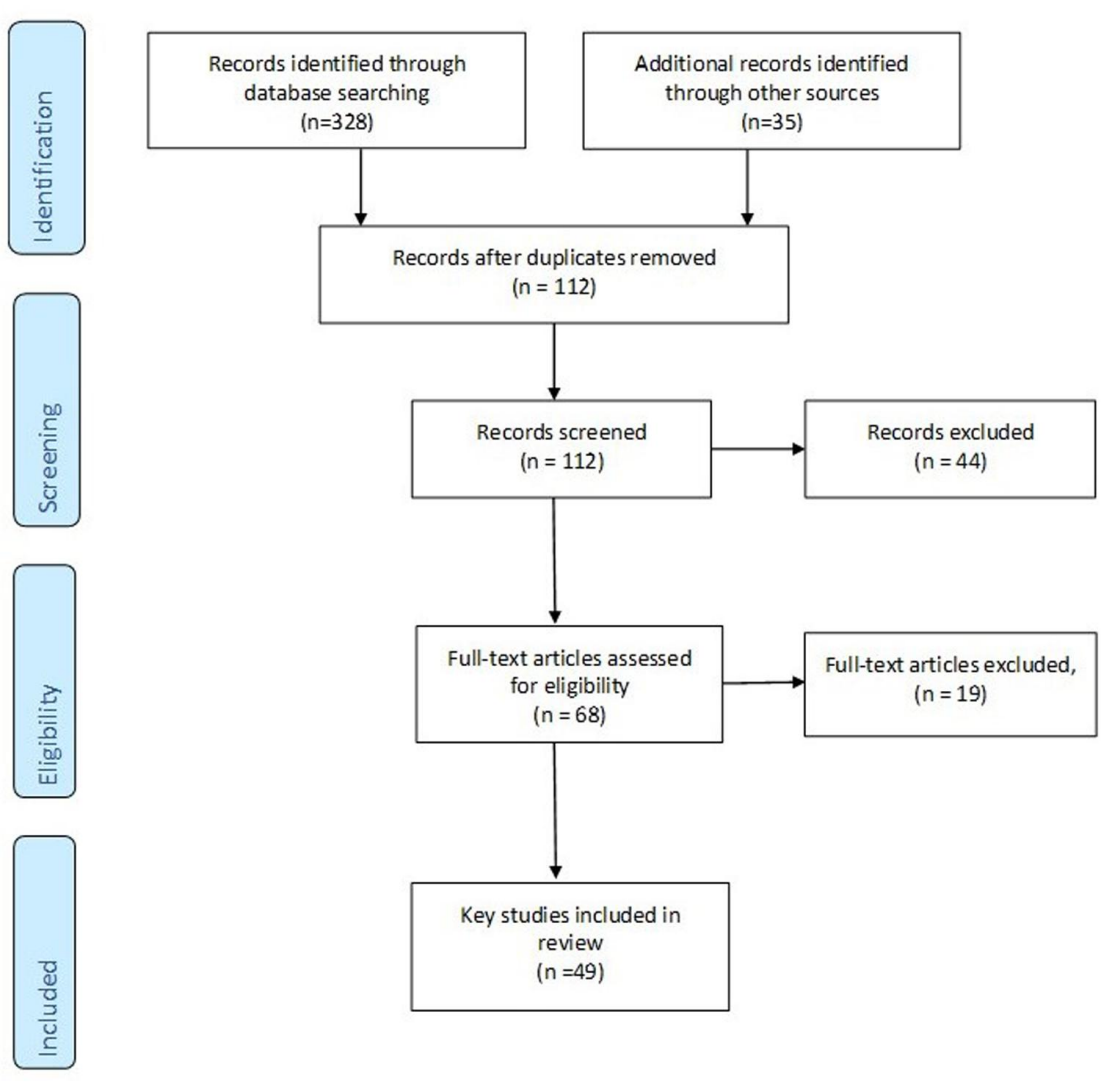
PRISMA diagram.

A total of 49 papers were selected for inclusion were included in the review. We initially retrieved 128 articles and after duplicates, protocols, or exclusion because they were not specific or relevant to one of the co-production steps or otherwise inclusive considerations were left with 49 papers explored in the review. The PRISMA Flow Diagram is shown in Figure 1.

The papers were authored across a total of 17 countries: the majority (22) were authored in Norh America [17 in the United States of America (USA), five in Canada], and 25 in Europe (including seven in the UK, and four in the Netherlands), with other countries including Australia and Malaysia. The work included consisted of reviews of various design, regulatory guidelines, white papers, commentaries, and primary research. A summary table of study characteristics can be found in Supplementary File 2.

### 3.1 Considerations to support inclusive digital co-production

Below we first reiterate the key principles of co-production as they pertain to the five core steps of the process. We then collate the current evidence by each of the five recognised steps of co-production.

#### 3.1.1 The principles of co-production

**Figure 2:**
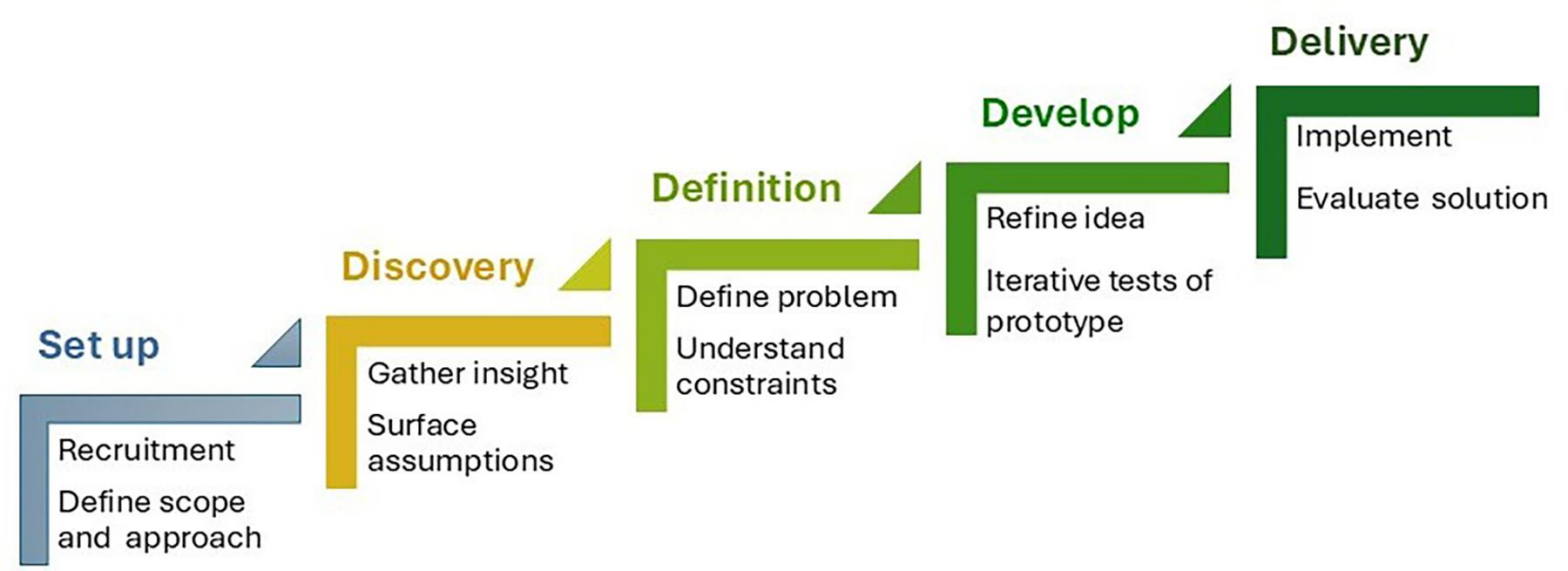
Five core steps of co-production.

A number of frameworks and methodologies have emerged to underpin co-production (35) and a recent systematic review of co-production in healthcare identified the same shared principles of various co-production approaches required in the democratic mobilisation of knowledge to improve health care and delivery including: bringing people together as active and equal partners, valuing all knowledge, using a creative approach, and iterative prototyping techniques (36). In operationalising these elements, all share versions of the same five core steps, namely: *Set-up*, this is where a range of participants are recruited reflective of all stakeholders, including user groups and broader community. It involves clear and collaborative agreement of the intended aims and outcomes of the process, including levels of involvement, the decision-making process, and the use of training to support the process and upskilling of participants; *Discovery*, where you gain an understanding of the context and issues at hand including exploring various perspectives, concerns and assumptions, and preferences and priorities; *Definition*, where the insights gained are prioritised, themes, patterns, and key problems are identified, and the feasibility of various solutions understood; *Development*, this is the opportunity to generate ideas and creative solutions. Early protypes might be developed to support learning through doing and help identify risks and previously unforeseen consequences. As ideas coalesce buy-in from external groups might be sought; and *Delivery,* the final step involves producing and launching the final solution, and map next steps and future sustainability. It is also an opportune moment to consider the performance and continuance of the co-production process (12, 37) Figure 2.

#### 3.1.2 The latest evidence in support of inclusive digital co-production

Below recent evidence of best practice in stakeholder engagement and digital health co-production is presented within each of the five key steps and constructs. This evidence is summarised in Table 1 and further explored below Table 2.

**Table 2:** Summary of considerations for inclusive digital co-production [after Man 2019 (37)].

Step	Constructs	Challenges	Approaches
Set-up	Recruitment	Patients: Overcoming barriers of opportunity or capacity to engage in co-design activities, lack of digital experience	Create hybrid design teams that include a diversity of experience within and between stakeholder groups (38) Allow time to address issues of awareness, opportunity, and resource necessary to contribute to co-production (39, 40) Encourage attendance by using flexible timing and location of sessions, and accessible venues (41). Consider time needed to raise awareness of digital health in underserved populations (40). Monitor inclusivity and representativeness of participants (age, ethnicity, disability, gender) (37)
		Staff: Recruit staff with a range of digital literacy; ensure they have time	Work closely with care organisations to identify staff with a range of roles, seniority and digital literacy/experience (42). Ensure there is ringfenced time and resource to allow for meaningful and continued staff contribution (43)
		Developers: Understand variety of size and maturity and experiences of co-production (in healthcare environments)	Describe the difference between user-centred design and co-production (44, 45). Understand the variation in digital partners from small start-ups to “big tech” (46). Ensure technology providers are aware of the importance of engaging underserved populations (47)
	Agree scope and approach	Accommodate diverse backgrounds and experience of digital systems	Provide training in co-production to empower all individuals, including, - raising awareness of the potential benefits of digital health, - confirm the democratic processes involved, and - the equitable importance of all voices (39, 48). Training should also explicitly describe the scope and approach of the initiative (49)
Discovery	Surface and challenge existing assumptions	Mistrust of mainstream healthcare organisations and security and confidentiality of digital health	Recognise the need for reassurance and trust-building with underserved populations (50, 51). Use culturally sensitive language and references (52–55)
		Uncertainty amongst health care providers including digital safety and changes in work practices	Explore safety concerns (56). Consider role of hybrid/analogue systems that retain patient contact (57) Address reservations amongst senior staff of meeting co-production recommendations for resource intensive solutions (58)
		Interaction between private sector and publicly funded healthcare	Funding model, profit and affordability, and intellectual property needs to be understood and agreed (59, 60) Balance patient preferences, clinical need, with affordability (and sophistication) of digital technology within spending constraints of healthcare system and patients (61, 62)
	Set priorities, and limitations	Surfacing and discussing contrasting agendas of patients, staff, and developers	Because brainstorming can favour those with more formal education, consider using more structured and iterative methods of ideation (19, 63–65)
Definition	Feasibility of solutions	Challenges of viability of digital solutions for patients	Explore contrasting needs of a diverse range of health and digitally literate patients (66)
		Staff capability, workflows and infrastructure	The fit of digital solution to existing workflows (67) and IT systems (68). The use of paper protypes to further develop ideas (69) The training requirements of staff expected to use the solution (70), and the use of digital champions to support implementation (71)
	Maintenance of engagement	Hybrid co-design team is resilient and functioning as intended	Monitor participants’ engagement in co-design, level of influence, transparency of decision-making, use tools like CUBE (72)
Development	Decide on idea(s) to take forward	Accommodate diverse expectations of what ’success’ looks like	Assess potential ideas aware of different criteria for each group of stakeholders, using equitable decision tools (37, 73–75)
	Steps towards implementation	The cost and complexity of piloting digital interventions	Test and gather feedback using cost-effective digital protypes: testing early and test often (28, 69, 76). The way in which the (pilot) solution meets regulatory requirements and evidence threshold (77, 78). Use structured implementation protocols (77, 79, 80)
Delivery	Future development, funding and support	What is needed to support sustainable roll-out of the intervention	Assess which steps needed for long-term integration and future innovation (81). Consider weight of evidence needed for take-up dependent upon the nature of the intervention (82)
	Establish long-term collaboration	Lack of evidence around maintaining long-term engagement in (digital) co-design	Evaluate co-design process (68) Including, - an understanding of the success of the process, - number of participants lost over time - - changes in participants confidence and skills, and facilitators and designers’ knowledge, and understanding of users (38, 83)

#### 3.1.3 Set-up

This first step consists of the recruitment of diverse stakeholders and the delineation of the scope and approach particular to the co-production initiative. In the context of digital health it has been recommended that hybrid co-production teams are created consisting of at least three diverse, stakeholder groups: patients and/or citizens, a range of senior decision-makers and providers from health and social care organisations, and digital health technology developers and suppliers from companies of various expertise, size, and maturity (38). These three groups are hereon referred to as “patients”, “staff” and “developers”.

There is consensus that the recruitment of patients or citizens to any healthcare related activity can be improved by addressing issues of awareness, opportunity, and resource (39, 40). To ensure engagement of all patient participants including underserved populations it is likely that some basic training in the core elements of digital health technologies is warranted (39). This might also include a lengthier period of formative design and development as the process continues to allow individuals to become adjusted to the concepts involved (40). Participation can be supported by flexible approaches in the timing, mode, and location of co-production activities, such as utilising community-situated venues and timing co-production activities to accommodate responsibilities of work and family (41). The payment of travel and expenses and the provision of vouchers is commonplace in patient engagement in health improvement activities, often guided by national bodies (84) but in the context of digital co-production this might usefully involve the provision of hardware or data packages to support familiarisation with digital technology. Finally, to ensure the intended diversity it is important to monitor the demographics of those enrolled (37).

The importance of enlisting a broad range of staff, representative of a range of digital experience, roles, and responsibilities, is understood: not only for the value of their individual experience, but also their subsequent influence on the acceptance of the digital solution amongst colleagues (42). There is evidence that it tends to be the digitally literate staff that enlist or support digital co-production with those less digitally inclined often overlooked (42). Similar to patients, to encourage the involvement of staff with limited digital experience some education may be needed as to the key concepts and capabilities of digital health. There are also more broadly recognised barriers to recruiting and retaining staff for healthcare initiatives relating to time and resource, with the understanding that meaningful participation can be encouraged by providing them with dedicated time and cover, acknowledging their participation as continuous professional development (43).

Arguably the least understood or explored group of stakeholders in digital co-production are those developing digital health technologies. Although many companies in the sector will be aware and have experience of user-centred design, the closely related process used in computer sciences (45); it is distinct from co-production in that understanding the needs of users occurs apart from the design process (whereas users are integrally involved in the design process in co-production) (44). The range of companies involved can range from non-profit social enterprise companies to international “Big Tech” such as Google and Palantir, with global interests in the use of data banks, aggregation platforms, and artificial intelligence (46). This can lead to significant variation in the understanding of patients and the healthcare environment, with similar differences in the resource and opportunity to engage in iterative co-production (47).

As with newly combined co-production teams in other domains of healthcare, this step should include the provision of training in what the process entails, the principles of equality and equitability, and the ask of their time and resource (48). This is also a timely opportunity for transparent and consensual agreement of the scope of the work to align the ambitions of those participating (49, 73).

#### 3.1.4 Discovery

The discovery step contains the discussion of challenges, assumptions and the setting of priorities for the co-production team. In a growing number of patients, particularly those from underserved populations this might involve addressing or overcoming issues of mistrust in centralised care organisations (41), as well as concerns around the security and confidentiality of digital health tools and data (50, 51, 85, 86). There is evidence that moderators might help to build trust by accommodating participants cultural backgrounds, primary languages, and cultural and faith practices (52–55).

Hesitancy towards digital health has also been reported amongst care providers, with concerns around the safety and reliability of digital health tools (56), with the recognition that moderators might start with an explicit discussion of the potential issues of using digital health tools (87). These include perceptions that digital solutions will reduce the in-person patient contact that many value and the role of hybrid/analogue or digitally-enhanced systems (38). There are also previously reported suspicions amongst senior-decision that co-production risks their commitment to potentially expensive or resource intensive solutions in order to meet emerging needs and preferences (58).

It is important that the potential funding model for the solution is discussed particularly for private sector technology developers, and commissioners where the additional cost can render digital solutions unsustainable (59). Although there are recommendations for the need for adaptable and value-based financing mechanisms, evidence of successful funding models is lacking (61, 88). Associated with this is the need to explore the expected profit margins (89), and a strategy agreed for where intellectual property lies (60), particularly in publicly funded and hybrid public/privately funded healthcare systems (61). These discussions require balancing what is desirable vs. what is affordable, not only to the health service but also patients, particularly underserved populations (62).

To support these initial conversations and potential solutions co-production initiatives typically use methods that involve abstraction and verbal communication such as open brainstorming (19). However, such approaches are more familiar to those exposed to formal education, and it has been suggested that carefully constraining the issue under discussion and using iterative sessions better suits a broader range of participants and provides more workable solutions (63, 64). This includes individual sessions structured around who is the target user, why they would use the tool/solution, the context in which they will use it, and early thoughts as to how the project team will gauge success of the solution (65).

#### 3.1.5 Definition

This step involves discussing the feasibility of the proposed solutions in terms of their fit, predominantly with patients and existing health services, though with implications for those developing and producing the technology (90). For underserved populations there are a number of well-rehearsed barriers to the take-up of digital health offers including access to hardware such as smart phones or PCs, the cost of data packages, and reliable internet connectivity and broader issues of digital and health literacy (66).

Health service representatives have a different set of constraints to consider. These relate to the characteristics on individual providers, their work practices, as well as the compatibility with existing care pathways and workflows (67). It is at this step where the training requirements of existing staff might be considered with acknowledged difficulties of self or experiential learning in delivering novel digital offers (70), and whether digital champions might be used to support patients and staff (71). This is also the point at which the impact on existing work processes must be understood as well as broader considerations of infrastructure including the necessary data assets, compatibility with existing IT systems, and the return on investment (68). These decision can be supported by paper prototyping, including sketches, diagrams, and storyboards are fast and inexpensive to create, and recommended for these earliest stages where the design direction is vague (69).

There are potentially difficult conversations to be had during this step where conflicting agendas are being aligned and difficult decisions being made, and there are recommendations that this is an appropriate time to understand the level of continued engagement across stakeholders. There are tools developed for this purpose that enable an understanding of levels of ownership, responsibility, and interactivity in the co-production team **(**37, 72).

#### 3.1.6 Development

This step involves deciding which idea or ideas should be taken forward and refined, alongside developing the plan for implementation. In making these choices it is important to recognise that each group of stakeholders will have varying priorities, and proposed solutions might be usefully scored on the different attributes valued by each (37), and categorised within the three domains of good design in healthcare; efficiency, safety, and usability (74). There are tools available designed to support equitable decision making amongst diverse groups which may be appropriate in this instance (91). These were borne of multi-criteria decision making that is more commonly understood within the field of operational research where alternatives are analysed with respect to a set of multiple (and often conflicting) criteria (75).

Refinement of the solution or tool requires digital rapid prototyping techniques are used to test more realistic and solid ideas; they should be realistic enough to accurately test most interface elements (69). They can be built using purposely developed prototyping tools and software (e.g., Marvel or Proto) or simple versions can also be made using presentation software like PowerPoint or Keynote (69). They are a cost-effective means of creating a prototype which would then be explored and further improved through a multi-phase (and pre-clinical) testing process (28, 76, 92, 93). Finally a high-fidelity prototype might be produced, and while valuable are also expensive and time-consuming and best used to refine near final versions or where complex interventions require accurate simulation (69).

A pre-determined time period of implementation should be agreed, with clearly defined roles and responsibilities of the stakeholders involved (78). A number of structured implementation frameworks for digital health have been developed (77, 79, 80). The implementation of novel digital health technologies is complicated by the intended functionality and identity of the intended user e.g., patients vs. providers) and it can evolve as they interact with surrounding system and processes (94). Though there is a lack of standardisation of which frameworks apply in which context, its recognised that they should involve a mixed method analysis of the impact, uptake, user experience and working mechanisms of the digital solution (77, 80) including a prior data-collection plan, agreed criteria for success or failure (78),.

#### 3.1.7 Delivery

The final step considers the long-term sustainability of the implementation, including assessing aspects of interoperability and integration with existing workflows, how well they support patient engagement and empowerment, and the degree to which the data collected can be used to evidence success and inform further innovation (68). In the UK the National Institute for Clinical and Health Excellence has created a useful three-tiered “Evidence Standards Framework” for digital health, where the level of evidence required to demonstrate effectiveness varies based on the technology's function and potential risk with one Tier 1 being the lowest (82). Specifically, these tiers are (1) Information and support tools (e.g., health tracking apps, symptom diaries); (2) technology used for health behaviour management, or preventative care; and the highest is Tier (3) Technologies that provide treatment or diagnosis (e.g., medical devices, digital therapeutics) (82).

It is also important to grasp the opportunity to learn more of which elements of co-production have proven most effective and how successfully a diverse range of stakeholders have been engaged (83). For example metrics might include the number of participants that remained throughout the process, or self-reported changes in their self-efficacy (38). This includes what moderators have learnt from the process, including their understanding of individual stakeholder groups and how they might be combined (83). Part of this process includes how the co-production dialogue can become continual, where previous co-production initiatives have been criticised for being short-term in their approach instead of continuing to provide feedback and providing tangible demonstration of the value placed on their time and input (95).

## 4 Discussion

Although the promise of digital co-production is apparent there are risks that health inequalities could be reinforced if the structural barriers that challenge the meaningful participation of diverse stakeholders remain unaddressed (96, 97). Previous examples of co-production that involve direct interaction between developers and underserved groups are rare (24). As we have described, the role of training and scope setting is key, both to manage expectations and to ensure consensual and transparent objectives. However, though there are several examples of regional training offers in the UK, there is no nationally or internationally recognised criteria for training in co-production (98).

The cost of digital solutions and the potential profits involved for private developers means it is particularly important that viable, cost-effective solutions that satisfy all parties are reached (61, 89). The inclusion of diverse populations in co-production might mean that the solutions proposed and developed have broader buy-in across diverse patient populations, it does not mean that they are independently capable of overcoming some of the social and environmental determinants of digital exclusion (99). These are without the power of health and social care organisations but should be coordinated, or otherwise supported by centrally mandated policies of national governments, such as subsidised coverage, limited data costs, and the use of open source software (39, 100). Such moves might be tempered by the lack of proven cost-effectiveness of digital interventions (101).

In thinking of the future for digital co-production, specific evidence relating to successful strategies is scarce with a lack of consensus over the metrics needed to support its long-term sustainability (102). This has led to calls for the reporting of (digital) co-production to be standardised to include two key perspectives: the impact on those participating e.g., whether they would remain involved or otherwise encourage others to participate, and the efficacy and sustainability of the outcomes (102). It is increasingly recognised that central bodies and health organisations should develop more flexible, and service specific approaches to promote a continuing co-production dialogue (81). This is particularly true when considering underserved and diverse patient populations where their involvement is likely their first interaction with co-production and may shape their ongoing relationship with health services (9, 103). This would ideally benefit from universal recommendations for digital inclusivity and the approaches that encourage it to be enshrined in policies that enables inclusive digital transformation and fosters innovation (104, 105). However, perhaps hindered by the paucity of evidence on best practice, policies specific to digital health co-production are yet to emerge.

Finally, it is worth noting that despite the majority of the work we have drawn upon being conducted in high-income countries digital health interventions in LMICs face many of the same barriers to digital health equity as those in HICs. These include inadequate infrastructure, limited digital literacy, regulatory challenges, lack of engagement, and high cost (106–109). Though there are as yet few examples of digital health co-production in LMICs, the potential of co-production to develop equitable care is increasingly being recognised (110). Although there may be heightened challenges to co-production in LMICs relating to more rigid hierarchical structures, socio-cultural beliefs, and political interference, there remains the potential for those interested to learn from the successful inclusive strategies highlighted here (111, 112).

### 4.1 Strengths and limitations

By placing our findings and recommendations in the context of the five core steps of co-production we have fulfilled our aim of producing a concise and coherent review of the challenges to inclusive co-production and how they might be overcome. The range of countries represented further demonstrates the international recognition of the value of co-production in a range of services including digital health. Though we would argue that the identified principles, tools and strategies that support inclusive co-production can be applied across multiple HICs and LMICs, we also acknowledge the value of tailoring initiatives to local populations and their particular sensitivities and needs. It is acknowledged that collective terms such as “underserved populations” as used here in reality describes a heterogenous group defined by socio-economic status, demographic characteristics and broader cultural factors (7). The implementation of any digital health co-production activity should necessarily accommodate the specific context and socio-cultural sensitivities of the target group (113).

### 4.2 Conclusions

With previous examples of co-production that involve direct interaction between developers and diverse groups of patients and staff rare, this integrative review has collated the latest evidence on engaging these diverse stakeholders in healthcare innovation, with best practice in co-production, and presents it within a framework representing the five core steps of co-production: *Set-up, Discovery, Definition, Development,* and *Delivery*. This guidance includes structured and tailored training in co-production and the concepts of digital health, surfacing and challenging existing assumptions around data security and confidentiality, defining funding models, introducing and refining protypes of increasing sophistication, and structured implementation and evaluation of both the co-production process and its outputs.
