# Supplementary material for: Increasing the inclusivity of digital health co-production: an integrative review
Source: Front Digit Health. 2025 Oct 17;7:1636469. doi: 10.3389/fdgth.2025.1636469 (PMC12575248; doi:10.3389/fdgth.2025.1636469)
Supplement: Supplementary file 1 [file Table1.pdf]

Supplementary Table 1 Characteristics of key studies since 2000 (n=49)

Core step	*Authors (year)*	*Country*	*Evidence type*	*Overview*	*Recommendations/comments*
1. Set-up	Avila Garcia et al 2019 [1]	USA	Primary research	Engaging users in mHealth	Low literacy and digital experience overcome by tailoring offer to individual user
	Badr et al 2022 [2]	Canada	Scoping review	Review and policy recommendations for digital inclusion	Align interventions with the needs of specific subgroups based on latest evidence
	Becker et al 2000 [3]	Netherlands	Conference paper	User involvement in human–computer interactions	Considers various practices in user-Centred Design including participatory Design and Socio-technical Design
	Browne et al 2022 [4]	Ireland	Primary research	Engaging health care professionals in research	Collaborate early at project concept and design stages. Create dedicated time and acknowledgement of participation as continuous professional development
	Chang et al 2004 [5]	USA	Conference paper	Reaching vulnerable populations with digital health	Recommendations include using partnerships, specific feedback to target populations
	Darejeh and Singh 2013 [6]	Malaysia	Review (unspecified)	How to develop digital interfaces	Considerations include reducing the number of features, and customizable font
	Duffy et al 2022 [7]	Canada	Narrative review	Implementing digital health design approaches	Collaboration of digital and health experts within hybrid design teams across all stages of intervention design
	Galvagno and Dalli 2014 [8]	Italy	Systematic review	The theory of co-production	Latest theories centre on service science, innovation and technology management, and marketing and consumer research
	Jagannathan et al 2020 [9]	USA	Review (unspecified)	Reconciling aspiration and outcome in co-production.	Progress from co-production is transformative but incremental

	Man et al 2019 [10]	USA	Guideline	Implementing and evaluating co-design.	Five key steps of co-design
	Richmond et al 2024 [11]	USA	Review (unspecified)	Building trust to bridge health inequity	Funding needed to support trust-related interventions with community members.<br>There is also a need to improve provider and system trustworthiness
	Schumacker et al 2023 [12]	Germany	Multiple case study	Analysis of Big Tech business model for digital health	Big tech is focussed on data driven preventive care, and diagnoses
	Zogas et al 2024 [13]	USA	Narrative review	Engaging patients in co-production	Challenges include limited funding for patient engagement exercises, and inadequate staff time
2. Discovery	Berry et al 2021 [14]	USA	Narrative review	Building trust -based partnerships in health care	Trust developed through empathy, discretionary effort, and fear mitigation.
	Borges et al 2023 [15]	Denmark	Review of reviews	Use of digital health technologies by health care professionals	Training, perception of usefulness and multi-stakeholder incentives enhance take-up
	Charle-Maachi et al 2022 [16]	France	Workshop paper	Value, funding mechanism and evaluation of digital interventions	Recommendations include shared and transparent methodology, early clarification of the technical scope of solutions and funding mechanisms.
	Chockshi and Mann 2018 [17]	USA	Review (unspecified)	Developing a process model to guide digital development	Recommends a structured process including design thinking and lean and agile approaches
	Harrington et al 2019 [18]	USA	Primary research	Equitable participatory design engagement	Need to look at historical context of research in target communities, improving access, and further consideration of unintentional harm of collecting personal narratives.
	Herlitz et al 2023 [19]	UK	Primary research	User experience of digitally enabled care	Need to accommodate patients with different needs; focus on the usability and interoperability of tech-enabled platforms

	Kotenko and Bohnhardt 2021 [20]	Ukraine	Comparative analyses	Financing digital health projects	Recommends establishing cooperation between the private, public sectors, academia, and non-governmental organizations
	Latonero and Aneja 2021 [21]	USA	Commentary	Co-design across public and private sectors	There should be mutual value across all actors, whether they are from civil society, the tech industry or government.
	Jones and Barry 2018 [22]	Ireland	Primary research	Generating trust in health promotion	Power-sharing and trust-building mechanisms need to be built into partnerships
	Lyles et al 2023 [23]	USA	Narrative synthesis	Digital health equity	Multi-pronged framework approach, taking into account individual and systemic factors
	Nandyal et al 2021 [24]	USA	Scoping review	Building community trust in secondary care	Effective understanding and communication with communities
	Smith 2022 [25]	UK	Scoping review	Future co-production priorities	Need to better explore outcomes, participants, inclusive language and costs
	Sparrey 2020 [26]	Canada	Book chapter	The use of structured brainstorming in co-production	Apply practical constraints to scope of individual discussions as part of an iterative process
	Steen et al 2018 [27]	Netherlands	Book chapter	Pitfalls in coproduction	Coproduction needs investment of time and money at national level
	Sultan et al 2019 [28]	USA	White paper	Cybersecurity awareness in underserved populations.	Lack of understanding of concept of cybersecurity, issues of language, and misplaced confidence in the security of their own cyber behaviours
	Tekic et al 2019 [29]	Russia	Commentary	Intellectual property in co-production	Can borrow from industry standards for deciding Intellectual property but needs to be contextualised (for digital health)
	Webb hooper et al 2023 [30]	USA	Primary research	Mistrust of healthcare in underserved populations	Use a community-responsive approach to provide lay-oriented education

3. Definition	Abernathey et al 2022 [31]	USA	Narrative review	Challenges of digital health transformation	Variety of recommendations for digital health inclusion
	Koru Ltd [32]	USA	Commentary	Design and use of rapid prototyping	Realistic enough to accurately test most interface elements.
	Litchfield et al 2021 [33]	United Kingdom	Rapid Review	Nature and impact of digital divide	Digital divide considered across three stages: Access, Literacy and Incorporation
	Longhini et al 2022 [34]	Italy	Systematic review	Digital health competencies among health care professionals	Embed in curricula, consider role of self-learning and experience
	Marwaha et al 2022 [35]	USA	Review (unspecified)	Adoption of digital health tools in large, complex health systems	Structured evaluation in advance of implementation
	Morieau et al 2021 [36]	Belgium	Primary research	Monitoring and measuring research engagement	A purposely designed tool for measuring engagement and decision making amongst participants
	Pettersen et al 2024 [37]	Norway	Systematic review	The role of champions in digital health adoption	Champions successfully promoted technology adoption
4. Development	Bate et al 2008 [38]	Netherlands	Book	Best practice in experience-based co-design	Concepts, methods and practices were discussed
	Ishizaka and Siraj 2018 [39]	United Kingdom	Primary research	Equitable decision making tools	Decision-making tools were useful even if followed inconsistently
	Kings Fund 2025 [40]	United Kingdom	Review (unspecified)	Designing inclusive and trusted digital health services with people and communities	Need to balance the power dynamics in coproduction groups, provide staff with support for sustained engagement

	Kip et al 2025 [41]	Netherlands	Primary research	Framework for Development, Implementation, and Evaluation of eHealth	Five structured phases including contextual enquiry, design, and evaluation
	Kushniruk et al 2006 [42]	Canada	Primary research	How medical error and workflow can inform improved safety	Simulation-based testing under a variety of conditions before the release of the final tool supports safety
	Mosch et al 2022 [43]	Germany	Primary research	Evidence based evaluation of digital health implementation	Need for pre-implementation assessment and continual evaluation and feedback
	National Health Service 2024 [44]	UK	Guidance	Piloting digital technologies in a health or care service.	Structured plan to fund implement and evaluate digital tech.
	Soobiah 2020 [45]	Canada	Review protocol	Optimal frameworks for evaluating/implementing digital health	Need to standardise frameworks to guide implementation of digital health care services
	Wallenius et al 2008 [46]	Finland	Primary research	Multiple criteria decision making	Need to expand engagement across occupations, and evaluate the effects of doing so
5. Delivery	National Institute for Health and Care Excellence 2022 [47]	United Kingdom	Regulatory guidance	Evidence standards framework for digital health technologies	A set of evidence standards for a wide range of digital tools to enable evaluators and decision-makers to identify likely benefits to users and system
	Pestoff 2014 [48]	Sweden	Review (unspecified)	Sustainability of citizen/user participation in co-production	Flexible, service-specific and organization-specific approaches for promoting co-production
	Von Huben et al 2023 [49]	Australia	Primary research	Funding of digital health technologies	Favoured criteria of funders include technical features, effectiveness, ethics, and economics

1. Avila-Garcia, P., et al., *Engaging users in the design of an mHealth, text message-based intervention to increase physical activity at a safety-net health care system.* JAMIA open, 2019. **2**(4): p. 489-497.
2. Badr, J., A. Motulsky, and J.-L. Denis, *Digital health technologies and inequalities: A scoping review of potential impacts and policy recommendations.* Health Policy, 2024. **146**: p. 105122.
3. Bekker, M. and J. Long. *User involvement in the design of human—computer interactions: some similarities and differences between design approaches*. in *People and Computers XIV—Usability or Else! Proceedings of HCI 2000*. 2000. Springer.
4. Browne, S., et al., *Reflections on recruiting healthcare professionals as research participants: Learning from the ONSPres Study.* HRB Open Res, 2022. **5**: p. 47.
5. Chang, B.L., et al., *Bridging the digital divide: reaching vulnerable populations.* J Am Med Inform Assoc, 2004. **11**(6): p. 448-57.
6. Darejeh, A. and D. Singh, *A review on user interface design principles to increase software usability for users with less computer literacy.* Journal of computer science, 2013. **9**(11): p. 1443.
7. Duffy, A., G.J. Christie, and S. Moreno, *The challenges toward real-world implementation of digital health design approaches: narrative review.* JMIR Human Factors, 2022. **9**(3): p. e35693.
8. Galvagno, M. and D. Dalli, *Theory of value co-creation: a systematic literature review.* Managing service quality, 2014. **24**(6): p. 643-683.
9. Jagannathan, K., et al., *Great expectations? Reconciling the aspiration, outcome, and possibility of co-production.* Current Opinion in Environmental Sustainability, 2020. **42**: p. 22-29.
10. Man, M., T. Abrams, and R. McLeod, *Implementing and evaluating co-design.* New Philanthropy Capital: London, 2019.
11. Richmond, J., et al., *Conceptualizing and Measuring Trust, Mistrust, and Distrust: Implications for Advancing Health Equity and Building Trustworthiness.* Annual Review of Public Health, 2023. **45**.
12. Schuhmacher, A., et al., *The dominant logic of Big Tech in healthcare and pharma.* Drug Discovery Today, 2023. **28**(2): p. 103457.
13. Zogas, A., et al., *Strategies for engaging patients in co-design of an intervention.* Patient Education and Counseling, 2024. **123**: p. 108191.
14. Berry, L.L., et al. *Trust-based partnerships are essential—and achievable—in health care service*. in *Mayo Clinic Proceedings*. 2021. Elsevier.
15. Borges do Nascimento, I.J., et al., *Barriers and facilitators to utilizing digital health technologies by healthcare professionals.* NPJ digital medicine, 2023. **6**(1): p. 161.
16. Charle-Maachi, C., et al., *What value do digital health solutions bring, what are the funding mechanisms and evaluations?* Therapies, 2022. **77**(1): p. 133-147.
17. Chokshi, S. and D. Mann, *Four phases for user-centered digital development: Integrating academic and industry approaches to health information technology.* JMIR Hum Factors, 2018.
18. Harrington, C.N., S. Erete, and A. Piper, *Deconstructing community-based collaborative design: Towards more equitable participatory design engagements. PACM on Human-Computer Interaction, 3, 216: 1-25*. 2019.
19. Herlitz, L., et al., *Patient and staff experiences of using technology-enabled and analogue models of remote home monitoring for COVID-19 in England: A mixed-method evaluation.* International Journal of Medical Informatics, 2023. **179**: p. 105230.

20. Kotenko, N.V. and V. Bohnhardt, *Digital health projects financing: challenges and opportunities.* 2021.
21. Latonero, M. and U. Aneja. *Co-designing Digital Interventions and Technology Projects with Civil Society [Internet]*. in *World Economic Forum*. 2021.
22. Jones, J. and M.M. Barry, *Factors influencing trust and mistrust in health promotion partnerships.* Global health promotion, 2018. **25**(2): p. 16-24.
23. Lyles, C.R., et al., *Multilevel determinants of digital health equity: a literature synthesis to advance the field.* Annual review of public health, 2023. **44**(1): p. 383-405.
24. Nandyal, S., et al., *Building trust in American hospital-community development projects: a scoping review.* Journal of community hospital internal medicine perspectives, 2021. **11**(4): p. 439-445.
25. Smith, H., et al., *Co-production practice and future research priorities in United Kingdom-funded applied health research: a scoping review.* Health Research Policy and Systems, 2022. **20**(1): p. 36.
26. Sparrey, C., *Learning Activity: Structured Brainstorming for the Co-production of Real-World Products*, in *Knowledge, Innovation, and Impact: A Guide for the Engaged Health Researcher: A Guide for the Engaged Health Researcher*. 2020, Springer. p. 187-190.
27. Steen, T., T. Brandsen, and B. Verschuere, *The dark side of co-creation and co-production: seven evils*, in *Co-production and co-creation*. 2018, Routledge. p. 284-293.
28. Sultan, A., *Improving cybersecurity awareness in underserved populations.* Center for Long Term Cybersecurity, UC Berkely. https://cltc. berkeley. edu/wpcontent/uploads/2019/04/CLTC_Underserved_Populations. pdf, 2019.
29. Tekic, A. and K.W. and Willoughby, *Configuring intellectual property management strategies in co-creation: a contextual perspective.* Innovation, 2020. **22**(2): p. 128-159.
30. Webb Hooper, M., et al., *Responding to healthcare distrust among underserved communities: phase II*. 2022, Wiley Online Library. p. 3-8.
31. Abernethy, A., et al., *The promise of digital health: then, now, and the future.* NAM perspectives, 2022. **2022**: p. 10.31478/202206e.
32. Koru. *The Role of Rapid Prototyping Techniques in Transforming Digital Healthcare*. 2025 [cited 2025 May]; Available from: https://www.koruux.com/blog/rapid-prototyping-techniques-in-healthcare/.
33. Litchfield, I., D. Shukla, and S. Greenfield, *Impact of COVID-19 on the digital divide: a rapid review.* BMJ open, 2021. **11**(10): p. e053440.
34. Longhini, J., G. Rossettini, and A. Palese, *Digital health competencies among health care professionals: systematic review.* Journal of medical Internet research, 2022. **24**(8): p. e36414.
35. Marwaha, J.S., et al., *Deploying digital health tools within large, complex health systems: key considerations for adoption and implementation.* npj Digital Medicine, 2022. **5**(1): p. 13.
36. Moriau, L., et al., *The engagement CUBE: a dialogical tool for designing, facilitating and monitoring engaged research and teaching strategies.* International Journal of Sustainability in Higher Education, 2022. **23**(4): p. 783-798.
37. Pettersen, S., H. Eide, and A. Berg, *The role of champions in the implementation of technology in healthcare services: a systematic mixed studies review.* BMC Health Serv Res, 2024. **24**(1): p. 456.
38. Bate, P. and G. Robert, *Bringing user experience to healthcare improvement: the concepts, methods and practices of experience-based design*. 2023: CRC Press.
39. Ishizaka, A. and S. Siraj, *Are multi-criteria decision-making tools useful? An experimental comparative study of three methods.* European Journal of Operational Research, 2018. **264**(2): p. 462-471.

40. Fund, T.K. *Designing inclusive and trusted digital health services with people and communities*. 2025; Available from: https://www.kingsfund.org.uk/insight-and-analysis/long-reads/inclusive-digital-services-people-communities.
41. Kip, H., et al., *The CeHRes Roadmap 2.0: Update of a Holistic Framework for Development, Implementation, and Evaluation of eHealth Technologies.* J Med Internet Res, 2025. **27**: p. e59601.
42. Kushniruk, A., et al., *Predicting changes in workflow resulting from healthcare information systems: ensuring the safety of healthcare.* 2006.
43. Mosch, L.K., et al., *Creation of an evidence-based implementation framework for digital health technology in the intensive care unit: qualitative study.* JMIR formative research, 2022. **6**(4): p. e22866.
44. NHS AI and Digital Regulations Service, *Piloting digital technologies in a health or care service*. 2024.
45. Soobiah, C., et al., *Identifying optimal frameworks to implement or evaluate digital health interventions: a scoping review protocol.* BMJ open, 2020. **10**(8): p. e037643.
46. Wallenius, J., et al., *Multiple criteria decision making, multiattribute utility theory: Recent accomplishments and what lies ahead.* Management science, 2008. **54**(7): p. 1336-1349.
47. Excellence, N.I.f.H.a.C. *Evidence standards framework (ESF) for digital health technologies*. 2022; Available from: https://www.nice.org.uk/about/what-we-do/our-programmes/evidence-standards-framework-for-digital-health-technologies.
48. Pestoff, V., *Collective Action and the Sustainability of Co-Production.* Public Management Review, 2014. **16**(3): p. 383-401.
49. von Huben, A., et al., *Stakeholder preferences for attributes of digital health technologies to consider in health service funding.* International Journal of Technology Assessment in Health Care, 2023. **39**(1): p. e12.
