# Supplementary material for: Increasing the inclusivity of digital health co-production: an integrative review
Source: Front Digit Health. 2025 Oct 17;7:1636469. doi: 10.3389/fdgth.2025.1636469 (PMC12575248; doi:10.3389/fdgth.2025.1636469)
Supplement: Supplementary file 2 [file Datasheet1.pdf]

Supplementary File 1 Sample of search terms

Underserved populations

"vulnerable populations"[MeSH Terms] OR ("vulnerable"[All Fields] AND "populations"[All Fields]) OR "vulnerable populations"[All Fields] OR ("underserved"[All Fields] AND "populations"[All Fields]) OR "underserved populations"[All Fields] OR "medically underserved area"[MeSH Terms] OR ("medically"[All Fields] AND "underserved"[All Fields] AND "area"[All Fields]) OR "medically underserved area"[All Fields]

Stakeholder engagement

"stakeholder participation"[MeSH Terms] OR ("stakeholder"[All Fields] AND "participation"[All Fields]) OR "stakeholder participation"[All Fields] OR ("stakeholder"[All Fields] AND "engagement"[All Fields]) OR "stakeholder engagement"[All Fields]

Digital health

"digital health"[MeSH Terms] OR ("digital"[All Fields] AND "health"[All Fields]) OR "digital health"[All Fields]

Coproduction

"coproduct"[All Fields] OR "coproduction"[All Fields] OR "coproducts"[All Fields]
